# RNA-seq revealed the effects of heat stress on different brain regions of *Leiocassis longirostris*

**DOI:** 10.3389/fphys.2025.1579499

**Published:** 2025-05-13

**Authors:** Senyue Liu, Qiang Li, Yongqiang Deng, Zhongwei Wang, Yang Feng, Han Zhao, Zhongmeng Zhao, Lu Zhang, Yuanliang Duan, Zhipeng Huang, Jian Zhou, Chengyan Mou

**Affiliations:** ^1^ Sichuan Fisheries Research Institute, Chengdu, Sichuan, China; ^2^ Institute of Hydrobiology, Chinese Academy of Sciences, Wuhan, China

**Keywords:** heat stress, brain, endoplasmic reticulum stress, lipid metabolism, *Leiocassis longirostris*

## Abstract

Understanding how distinct brain regions of *Leiocassis longirostris* molecularly adapt to heat stress is vital for improving aquaculture sustainability and guiding conservation strategies in a warming climate. To elucidate the region-specific molecular mechanisms underlying heat stress responses in the brain of *L. longirostris*, we exposed L. longirostris to acute heat stress (32°C) for 24 h and performed RNA-seq and WGCNA on five brain regions (OB: olfactory bulb, FB: pituitary, hypothalamus, forebrain, MB: mesencephalon, CB: cerebellum, and SC: spinal cord). The results showed that, after heat stress, the FB region significantly activated the ER stress pathway, and the abnormal proteins were synergically cleared by HSP-mediated UPR (such as Hsp70, Hsp90, IRE1α, Perk, ATF6) and UPS-mediated ERAD (such as UBE2, UBE3, TRIM63). Meanwhile, the SC region showed marked downregulation of lipid metabolism and PPAR signaling pathway, suggesting energy conservation as a compensatory strategy. WGCNA further highlighted the FB as the hub for ER stress and the SC for metabolic suppression. In conclusion, our study suggests that distinct brain regions of *L. longirostris* adopt different strategies under heat stress, in which the FB region mediates protein quality control and the SC region drives metabolic inhibition. These findings highlight the adaptation strategies of the *L. longirostris* brain to heat stress and provides a potential target for improving its survival under global warming.

## 1 Introduction

As poikilotherms, fish exhibit body temperatures that equilibrate rapidly with ambient water, making temperature a pivotal abiotic factor governing their physiological and biochemical processes (Beitinger et al., 2000). Climate change-induced temperature extremes, such as heatwaves, pose significant threats to aquatic ecosystems (Somero and Hochachka, 1971). Previous studies have mainly focused on the effects of heat stress on the gills and intestines of fish (Dawood et al., 2022; Sun et al., 2015). However, the brain, which serves as the central hub for sensing environmental changes and coordinating systemic adaptations, has been relatively understudied, especially how different brain regions react at the molecular level.

The vertebrate brain is anatomically and functionally compartmentalized. In fish, the brain is divided into five regions (OB: olfactory bulb, FB: pituitary, hypothalamus, forebrain, MB: mesencephalon, CB: cerebellum, and SC: spinal cord) (Wang et al., 2021) with different physiological functions. For example, regions such as the hypothalamus (located in the FB region) regulate stress response and homeostasis (Uchimura et al., 2019), while the SC region regulates metabolic activity (Grillner, 2021). However, whether distinct brain regions employ specialized strategies to counteract heat stress, and how these strategies interact to ensure survival, are critical questions yet to be resolved. Addressing these gaps is essential for understanding the evolutionary adaptations of fish to warming environments and for mitigating aquaculture losses caused by rising temperatures.

*Leiocassis longirostris*, one of the most economically valuable freshwater fish in China, thrives at 25°C–28°C but faces survival challenges beyond 32°C (He et al., 2021). Previous studies on this species have primarily examined gill and intestinal damage under acute heat stress (Zhao et al., 2024), leaving its neural adaptation mechanisms poorly characterized. Exploring how heat stress affects different brain regions in *Leiocassis longirostris* will deepen our comprehension of its metabolic physiology and stress response, and also offer insights into how this species evolves to cope with temperature change.

In this study, the transcriptomic responses of five brain regions of *L*. *longirostris* under heat stress were systematically analyzed to elucidate the specific molecular adaptation mechanisms and their synergistic effects. Our findings not only advance the understanding of heat-adaptation mechanism of *L. longirostris*, but also identify potential targets for enhancing thermal adaptation in aquaculture species, which is a pressing need in the context of global climate change.

## 2 Materials and methods

### 2.1 Experimental fish

As previously described (Zhao et al., 2024), the *L*. *longirostris* used in this study came from the Sichuan Fisheries Research Institute. Fish that were 3 months old, had similar sizes (10.45 ± 0.26 cm; 18.32 ± 1.11 g), showed normal swimming behavior and the absence of any physical injuries were selected as the experimental fish. Before the formal experiment, fish were temporarily raised for 1 week in a round breeding tank under specific environmental conditions. The water temperature was 26°C ± 0.5°C, the dissolved oxygen ≥6.5 mg L^−1^ and pH = 7.4 ± 0.14. One-third of the water was changed daily, and the fish were feed twice (9 a.m. and 9 p.m.).

### 2.2 Heat stress experiment and sample collection

**FIGURE 1:**
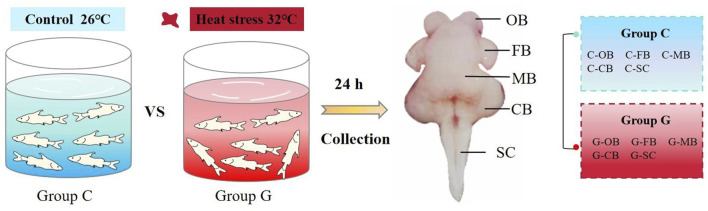
Model diagram of experimental treatment OB, olfactory bulb; FB, pituitary, hypothalamus, forebrain; MB, mesencephalon; CB, cerebellum; SC, spinal cord. Refer to Wang et al. (2021).

*L. longirostris* exhibits an optimum growth temperature range of 25°C–28°C (He et al., 2021). Therefore, fish in this study were divided into two groups, the heat stress group (32°C, group G) and the control group (26°C, group C). Each group comprised three replicates, and within each replicate, there were ten fish. Subsequently, the two groups of fish were subjected to acute heat stress for 24 h under the predefined temperature conditions (Figure 1).

After 24 h of heat stress, one fish was randomly selected from each of the three parallels in the two temperature treatment groups, and anesthetized with buffered MS222 (250 mg L^−1^, Aladdin, China). And according to the study of Wang et al. (2021), the brain was further divided into five distinct regions (OB, FB, MB, CB and SC). After collecting the brain region samples of the two groups (a total of 30 samples, three samples obtained from each brain region within both Group C and G group), these samples were promptly stored at −80°C for downstream analysis.

### 2.3 Total RNA extraction

Total RNA was extracted from 30 brain samples using Trizol reagent (Takara Bio, Japan) according to the manufacturer’s instructions. Nanodrop2000 was used to detect the concentration and purity of the RNA, agarose gel electrophoresis was used to detect the RNA integrity, and Agilent 2100 was used to determine the RIN value. Samples meet the standards were used for subsequent test (Total RNA ≥1 μg, RNA concentration ≥45 ng μL^−1^, 1.8 ≤ OD260/280 ≤ 2.2, 2.0 ≤ OD260/230 ≤ 2.2). After total RNA was extracted, eukaryotic mRNA was enriched by Oligo(dT) beads.

### 2.4 Transcriptomics (RNA-seq) analysis

#### 2.4.1 Library preparation and sequencing

The RNA-seq was performed by Omicsmart (Guagzhou, China) using the Illumina NovaSeq6000 platform. To ensure the quality of the data and the accuracy of subsequent analysis outcomes, the software FastQC was employed to conduct quality control on the original sequencing data. The RNA-seq data generated in this study is publicly accessible in the National Center of Biotechnology Information (NCBI) database, with the accession numbers PRJNA1209670 and PRJNA1211834.

#### 2.4.2 Sequence reads mapping

The high-quality mRNA reads were mapped with the reference genome (GDR21070358-2 _std_1) to obtain mapped data (reads) for subsequent analysis. HISAT2 software (https://github.com/DaehwanKimLab/hisat2) was used for sequence alignment analysis. And software Cufflinks (http://cole-trapnelllab.github.io/cufflinks/) was used to assemble the mapped reads (Kim et al., 2019).

#### 2.4.3 Functional annotation and sample relationship analysis

All transcripts and corresponding genes were compared for functional annotation and classification with the Gene Ontology (GO), Kyoto Encyclopedia of Genes and Genomes (KEGG) databases. Principal component analysis (PCA) was performed with R package gmodels (http://www.r-project.org/) in this experience.

#### 2.4.4 Analysis of differentially expressed genes (DEGs) and functional enrichment

RNAs differential expression analysis was performed by DESeq2 software (Love et al., 2014) among five groups (C-OB vs. G-OB, C-FB vs. G-FB, C-MB vs. G-MB, C-CB vs. G-CB and C-SC vs. G-SC). To account for the false discovery rate (FDR), P-values were modified using the Benjamini–Hochberg approach (Madar and Batista, 2016). Specifically, the thresholds for identifying significant differential expression were set as |log2FC| > 1 and p adjust < 0.05.

### 2.5 RNA-seq data analysis

The raw RNA-seq data obtained by sequencing were quality controlled using fastp to obtain clean read segments. The transcript read counts were calculated for each sample using RSEM. The number of read counts in each sample was normalized and transcript expression levels were analyzed using the DESeq2 software package.

### 2.6 Gene set enrichment analysis (GSEA)

Gene set enrichment analysis was performed by R software package (Subramanian et al., 2005) to identify whether a set of genes in specific GO terms\KEGG pathways shows significant differences in two groups. Briefly, the gene expression matrix was input, and the genes were ranked by SignaltoNoise normalization method. Enrichment scores and p value was calculated in default parameters.

### 2.7 Weighted gene co-expression ntwork analysis (WGCNA)

The Weighted Gene Co-expression Network Analysis (WGCNA) of *L*. *longirostris* brain was performed using R packets, with the aim of exploring the core genes within the network. Expression matrix transformation of transcriptome data was performed using Variance Stabilizing Transformation (VST). The appropriate soft thresholding power was determined according to the scale-free network principle, and the gene co-expression network was constructed. Topological overlap degree (TO) was used to characterize the correlation degree among genes, and the adjacency matrix was transformed into topological overlap matrix (TOM). Subsequently, module identification was performed by applying the dynamic tree cut method, with 1 - TOM being utilized as the gene clustering distance (Langfelder and Horvath, 2008; Chen et al., 2022). To identify significant modules, the correlation between the expression of heat acclimation marker HSF (heat shock transcription factors) (Gomez-Pastor et al., 2018; Rao et al., 2022) and the modules was calculated. Finally, the regulatory network was visualized through the application of Cytoscape software.

### 2.8 Ethical approval

All animal handling procedures were approved by the Animal Care and Use Committee of the Fisheries Research Institute, Sichuan Academy of Agricultural Sciences (Chengdu, China), following the recommendations in the ARRIVE guidelines, under permit number 20210307001-5. At the same time, all methods were carried out by relevant guidelines and regulations.

## 3 Results

### 3.1 Evaluation of transcriptome sequencing data

Transcriptome sequencing of 30 samples was performed using the Illumina NovaSeq6000 platform. Raw reads were subjected to quality control in fastp format, and low-quality data were filtered to obtain clean reads. After the data was filtered, the base composition and mass distribution were analyzed to visually display the data quality. The Sequencing data quality table is presented in Supplementary Table S1. The results of mapping comparison with reference genomes are shown in Supplementary Table S2, and the mapping rate (Total Mapped) was higher than 88.14%.

### 3.2 Sample relationship analysis and basic differential gene analysis

**FIGURE 2:**
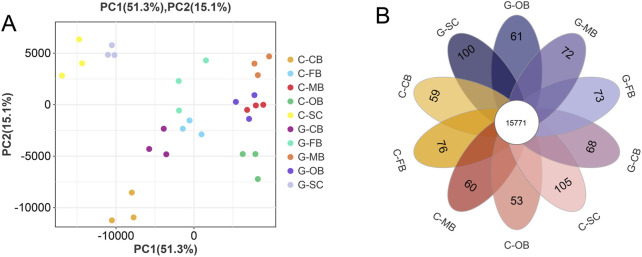
Relationship analysis of five brain regions samples **(A)** Principal component analysis (PCA) of five brain regions. **(B)** The intersample Venn diagram shows common genes and specific genes between groups. The numbers in the overlapping regions represent the number of common genes between the groups, and the numbers in the non-overlapping regions represent the genes unique to each group.

**FIGURE 3:**
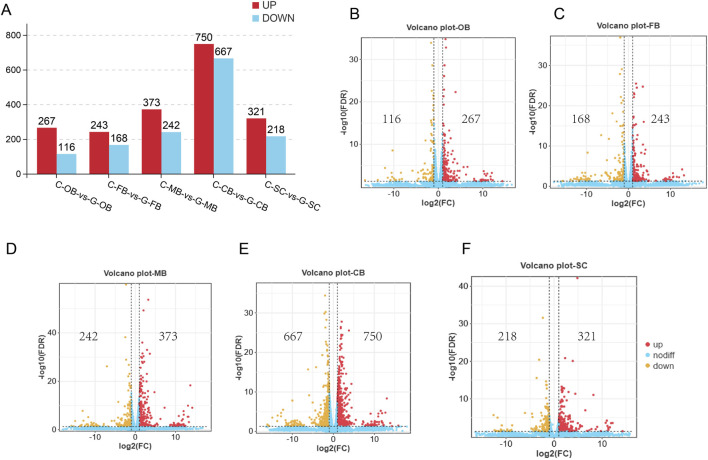
Analysis of basic differences in five brain region samples **(A)** The number of DEGs that met the threshold screening in each group. **(B–F)** The volcano plot shows the distribution of DEGs between C-OB vs. G-OB, C-FB vs. G-FB, C-MB vs. G-MB, C-CB vs. G-CB and C-SC vs. G-SC, respectively. Red dots indicate upregulated DEGs, orange dots indicate downregulated DEGs, and blue dots indicate genes with no difference.

To analyze the repeatability among samples, we conducted PCA based on the expression level. The outcomes demonstrated that 30 samples (three samples obtained from each brain region within both Group C and G group) were clustered into separate and independent groups respectively (Figure 2A). According to Venn analysis (Figure 2B), there were a large number of common genes and unique genes among the 10 groups. As shown in the Figure 3, a total of 383 DEGs, 411 DEGs, 615 DEGs, 1,417 DEGs, and 539 DEGs were obtained between C-OB vs. G-OB, C-FB vs. G-FB, C-MB vs. G-MB, C-CB vs. G-CB and C-SC vs. G-SC, respectively.

### 3.3 GO enrichment analysis of DEGs in brain tissue

**FIGURE 4:**
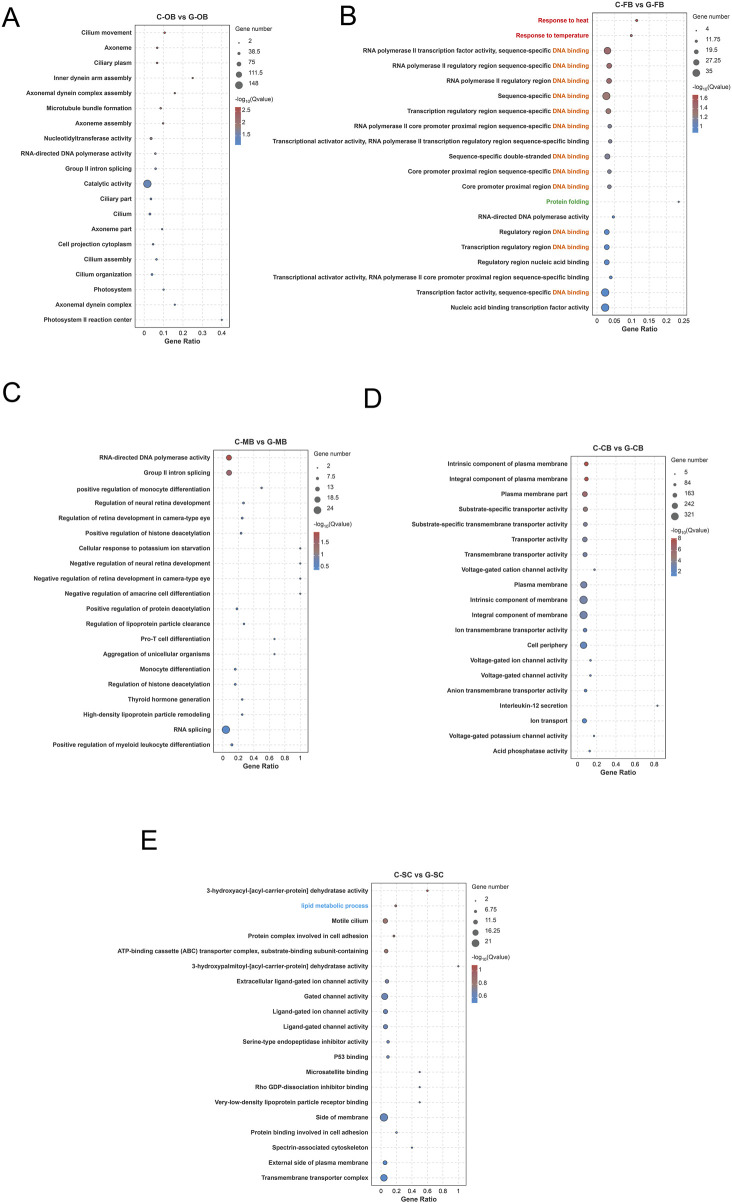
GO enrichment analysis of DEGs in different brain regions of *Leiocassis longirostris* after heat stress **(A–E)** GO enrichment analysis of DEGs between C-OB vs. G-OB, C-FB vs. G-FB, C-MB vs. G-MB, C-CB vs. G-CB and C-SC vs. G-SC, respectively. The vertical axis represents the name of the pathway, and the horizontal axis Rich factor represents the ratio of sample number/background number. The size and color of the dots represent the number of genes and the P adjust of each pathway, respectively.

To further explore the function of DEGs, all DEGs were mapped to GO term encompassed within the GO database for enrichment and classification. These GO terms were categorized into three main aspects, including molecular function (MF), cellular component (CC), biological process (BP) as shown in Supplementary Figure S1. According to the enrichment results of the top 20 items presented by GO enrichment analysis (Figure 4), the GO functions enriched between C-FB and G-FB (Figure 4B) mainly included response to heat, response to temperature, protein folding, and DNA binding. This outcome implies that the FB region serves as a major region responsible for responding to thermal stimulation and might be closely associated with protein synthesis and folding under high-temperature stimulation conditions. Furthermore, the Lipid metabolic process was found to be enriched in C-SC vs. G-SC (Figure 4E), suggesting that the SC region may play a role in regulating lipid metabolism within the brain of *L. longirostris* under heat stress.

### 3.4 KEGG enrichment analysis of DEGs in brain tissue

**FIGURE 5:**
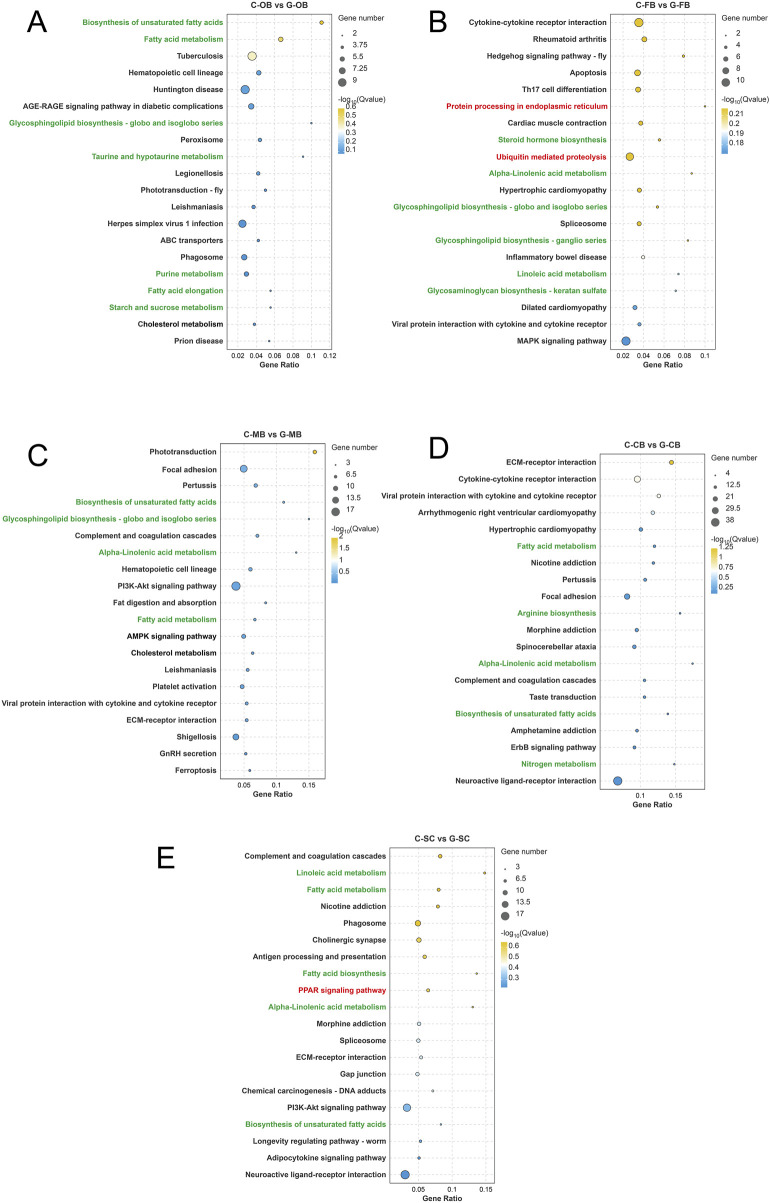
KEGG enrichment analysis of DEGs in different brain regions of *Leiocassis longirostris* after heat stress **(A–E)** KEGG enrichment analysis of DEGs between C-OB vs. G-OB, C-FB vs. G-FB, C-MB vs. G-MB, C-CB vs. G-CB and C-SC vs. G-SC, respectively.

In order to further explore the biological processes involved in DEGs, we mapped all the transcripts of DEGs into the KEGG database to enrich and classify them, and the results were presented in Supplementary Figure S2. According to the top 20 enrichment KEGG pathways (Figure 5), metabolism-related pathways were widely enriched across all the five brain regions. These pathways were mainly related to fatty acid and amino acid metabolism, such as fatty acid metabolism, biosynthesis of amino acids, alpha-linolenic acid metabolism, and fatty acid biosynthesis.

Moreover, C-FB vs. G-FB (Figure 5B) enriched Protein processing in endoplasmic reticulum (ER), and Ubiquitination-mediated proteolysis. This indicates that the FB region may repair misfolded proteins by activating the ER protein processing, and meanwhile degrade abnormal proteins that cannot be repaired through the ubiquitination mechanism, so as to maintain the body’s homeostasis.

It is worth noting that, the SC region showed reduced activity in the PPAR pathway (Figure 5E), a critical regulator of fat production, indicating that SC region plays a central role in controlling lipid metabolism during heat stress. Together, these results suggest that different brain regions underwent different molecular and biological processes during heat stress.

The vertical axis represents the name of the pathway, and the horizontal axis Rich factor represents the ratio of Sample number/Background number. The size and color of the dots represent the number of genes and the P adjust of each pathway, respectively.

### 3.5 Effects of heat stress on heat response and protein processing in brain

As numerous studies (Kopp et al., 2019; Hwang and Qi, 2018) have demonstrated that heat treatment has a significant impact on protein synthesis and folding, we have selected the key genes and signaling pathways related to heat response, ER protein processing and folding, and ubiquitination-mediated proteolysis for analysis.

**FIGURE 6:**
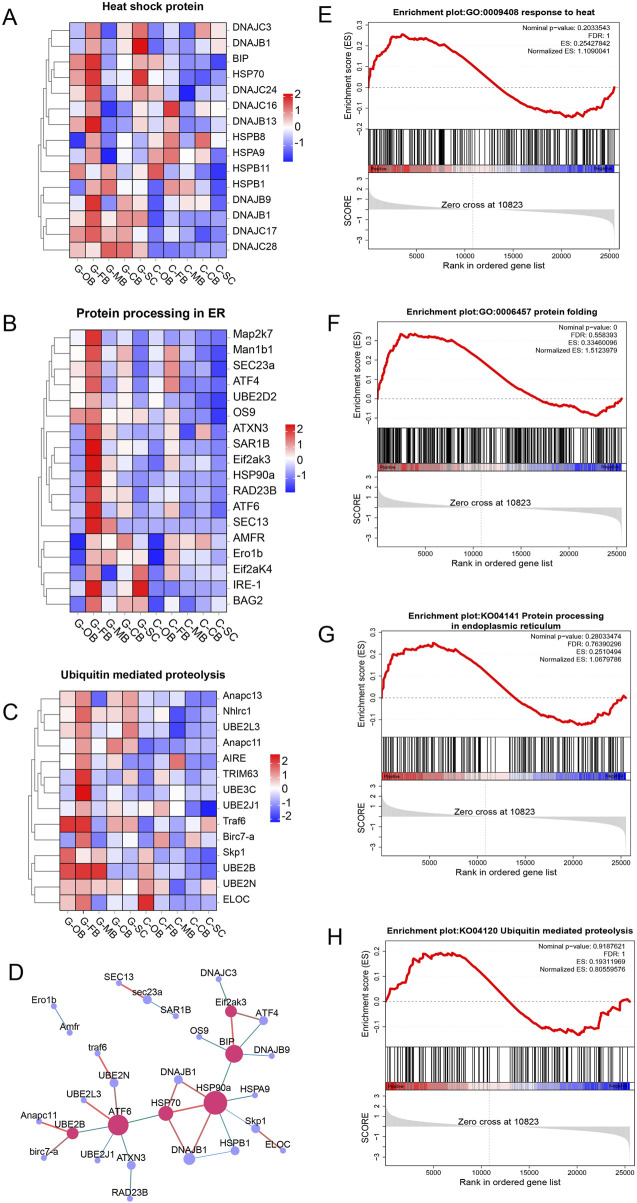
Effects of heat stress on heat response and protein processing in brain of *Leiocassis longirostris* **(A–C)** Hierarchical clustering analysis based on FPKM of DEGs related to HSP, protein processing in ER and ubiquitination mediated proteolysis, respectively. Red and blue indicated that the gene expression level was upregulation and downregulation, respectively. **(D)** PPI networks after heat stress. **(E–H)** GSEA analysis of the FB region for heat response, protein folding, protein processing in ER and ubiquitination mediated proteolysis, respectively.

As shown in the results, compared with the control group, the expression of heat shock protein (HSP) genes was significantly upregulated across all five brain regions within Group G (Figure 6A), especially the FB region. GSEA analysis conducted using the GO database revealed that the heat response pathway in the FB region was markedly upregulated (Figure 6E). These results indicated that the FB region widely expressed HSP-related genes and actively engages in responding to heat stress under high-temperature conditions.

In the analysis of protein processing, we found that, after heat stress, genes related to ER mediated protein folding and processing (Figure 6B), as well as genes associated with protein ubiquitination degradation (Figure 6C) were observably higher in the FB region. Moreover, GSEA analysis revealed that three pathways including protein folding (Figure 6F), protein processing in endoplasmic reticulum (Figure 6G) and ubiquitination mediated proteolysis (Figure 6H) were also significantly upregulated in the FB region. In light of the above findings, we hypothesized that the FB region induces ER stress under heat stress.

Through the mapping of the Protein-protein interaction (PPI) networks based on the protein interaction relationship (Figure 6D), it was found that under high-temperature conditions, the coordination between HSP and ubiquitin ligase can promote protein processing within the ER and contribute to the removal of abnormal proteins from the body, among which *UBE202*, *ATF6*, *Hsp70*, *Hsp90a*, *Eif2ak3*, *BIP* and *DNAJ (Hsp40)* were identified as key genes.

### 3.6 Effects of heat stress on lipid metabolism in brain

Since we found that metabolism-related processes, especially lipid metabolism, were widely enriched across the five brain regions, we hypothesized that heat stress strongly affects the regulation of lipid metabolism within the brain of the *L. longirostris*.

**FIGURE 7:**
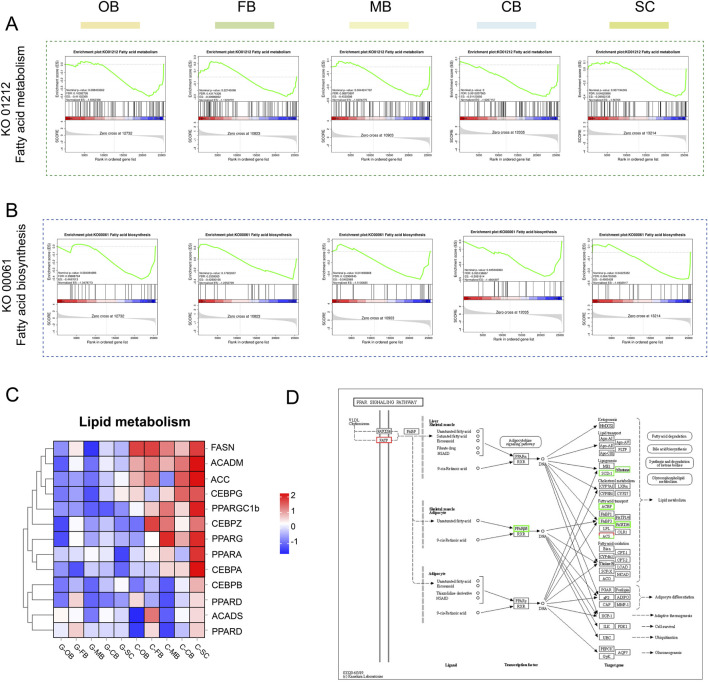
Effects of heat stress on lipid metabolism in brain of *Leiocassis longirostris* **(A, B)** GSEA analysis of fatty acid metabolism and fatty acid biosynthesis, respectively. **(C)** Hierarchical clustering analysis of DEGs related to lipid metabolism. **(D)** PPAR signaling pathway of SC region.

For further study, the fatty acid metabolism (Figure 7A) and fatty acid biosynthesis (Figure 7B) pathways were selected for GSEA analysis. The results showed that the whole brain region expression of group G was downregulated compared with group C. Subsequently, key genes involved in lipid metabolism were selected for cluster analysis (Figure 7C), and the results showed that most of the lipid metabolism-related genes in the whole brain region were downregulated after high temperature stress. Among them, the downregulation of SC region was the most obvious (Figure 7C). Moreover, the PPAR signaling pathway, a key regulator of lipid metabolism, was found to be significantly downregulated in C-SC vs. G-SC (Figure 7D).

Collectively, lipid metabolism in the brain of *L. longirostris* was inhibited under high-temperature stress, and the SC region was the key target region. By reducing the consumption of oxygen and energy, this inhibition process can achieve a compensatory regulatory effect on energy metabolism disorders caused by high temperature stress.

### 3.7 Weighted gene co-expression network analysis (WGCNA)

**FIGURE 8:**
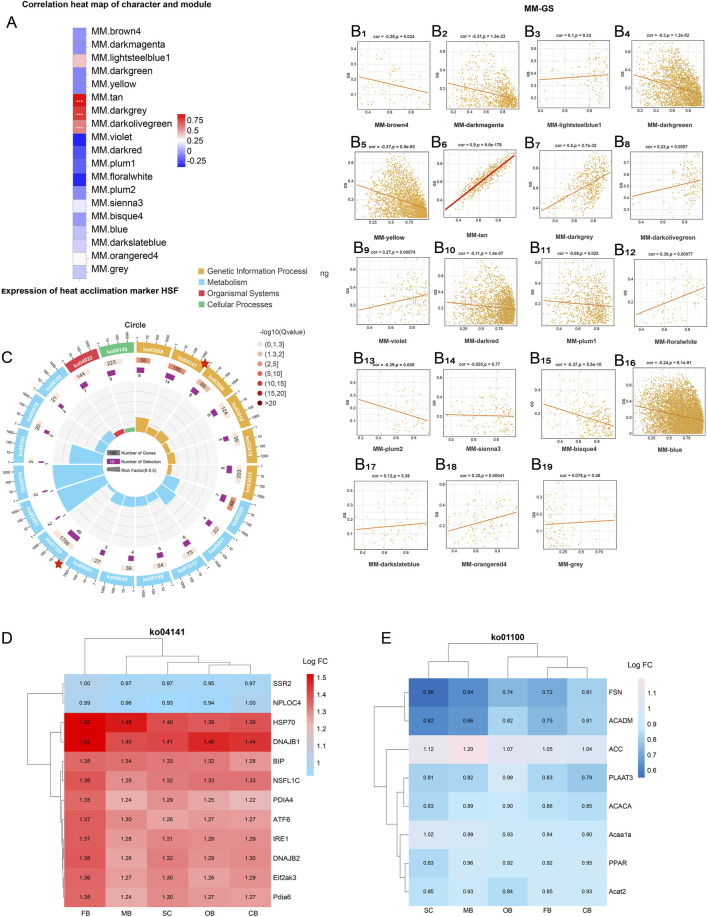
WGCNA after heat stress **(A)** correlation analysis of characters and modules. **(B)** MM-GS correlation analysis. **(C)** KEGG enrichment analysis of Module tan. **(D)** Cluster analysis of the key genes in ko 04141. **(E)** Cluster analysis of the key genes in ko 01100.

In order to find the co-expressed gene modules, explore the relationships and networks among various genes, as well as find the hub gene, we used WGCNA to analyze DEGs. A total of 19 modules with related expression patterns were clustered together (Supplementary Figure S3). Through the correlation analysis between characters and modules (Figure 8A), as well as the MM-GS analysis (Module Membership and Gene Significance, Figure 8B), we found that the Module tan was most closely related to heat stress.

Subsequently, genes in Module tan were mapped to the KEGG database for enrichment and classification, with Ko 04141 (protein processing in endoplasmic reticulum) and ko 01100 (metabolic pathway) were the most representative pathways (Figure 8C). Cluster analysis of key genes of Ko 04141 showed an upregulation trend in all five brain regions. Among them, the FB region was the area with the most significant change, and HSP70 and DNAJB1 (HSP40) were the key regulatory genes with the most obvious changes (Figure 8D). Different from Ko 04141, the key genes of Ko 01100 were downregulated in all five brain regions. The downregulation of SC region was the most significant, and FSN and ACADM were the most critical genes (Figure 8E).

## 4 Discussion

### 4.1 Brain regions exhibit distinct thermoregulatory roles

In this study, GO and KEGG analyses revealed specialized molecular responses across *L. longirostris*’ brain regions. After heat stress, the FB region (hypothalamus-containing region) emerged as the central hub for heat stress adaptation, marked by upregulation of heat response, protein processing in endoplasmic reticulum, and ubiquitination-mediated proteolysis (Figures 4, 5). Therefore, we hypothesized that, the FB region maintain homeostasis through timely repair and degradation of damaged proteins, which is consistent with the widely reported function of the hypothalamus as a receptor for external temperature stimulation (Duan and Xu, 2019).

The SC region is located in the ventral part of the cerebellum and extends backward. It serves as the center of various life activities, such as controlling cardiovascular activities and regulating metabolism (Grillner, 2021). In this study, after high-temperature stress, the SC region showed pronounced suppression of lipid metabolism (Figures 7C, D), including downregulated PPAR signaling pathway and genes related to fatty acid biosynthesis and fatty acid metabolism. This indicates that the SC region is a key region responsible for regulating fatty acid metabolism following high-temperature stress.

### 4.2 ER stress is a hallmark of heat-stressed brain tissue

ER is a large and intricate network composed of complex membranes, with approximately one-third of protein production and folding taking place within this organelle (Hebert and Molinari, 2007). Inside ER, secretory and membrane proteins undergo folding, modification, and assembly procedures to form their final functional protein structures. The release of such proteins from ER is precisely regulated, and only properly folded proteins can be expelled from ER (Wickner and Schekman, 2005). Nevertheless, given that protein folding is a complex and error-prone process, the protein folding capacity of ER is easily saturated under certain physiological conditions or environmental stresses (including nutrient deficiency, hypoxia, and high temperature), leading to ER stress (Mao et al., 2019).

Numerous studies have shown that heat treatment has a significant impact on protein synthesis and increases chaperon-mediated protein folding, ultimately inducing ER stress (Ma et al., 2022; Chen et al., 2023). In this study, through WGCNA we identified Module tan as heat-responsive, with key genes (*HSP70*, *DNAJB1*) linked to ER protein processing (KEGG:04141, Figure 8D). And the endoplasmic reticulum stress processes such as UPR and ERAD were significantly activated in the FB (Supplementary Figure S4). Those align with ER’s role in managing misfolded proteins under thermal stress, confirming FB as the primary site for ER stress mitigation in *L. longirostris*’ brain.

### 4.3 The regulatory mechanism of endoplasmic reticulum quality control under heat stress

Under heat stress conditions, cells respond to ER stress induced by misfolded protein accumulation mainly through ER unfolded protein response (UPR) (Buchberger et al., 2010) and ER associated degradation (ERAD) (Li et al., 2017). In this study, three ER stress receptors/transmembrane proteins (*IRE1*, *EIF2AK3*, *ATF6*) (Deng et al., 2011) were significantly expressed in the FB region after high temperature treatment (Figure 6B), indicating that this region is the core region involved in ER stress. Heat shock protein (HSP), as a major ER chaperone (Park and Park, 2019), helps repair damaged proteins by promoting proper protein folding and mediating UPR (Cheng et al., 2015; Han et al., 2025) In this study, after heat stress, HSP protein-related genes showed an upregulation trend in the whole brain region (Figure 6A), indicating that HSP helped repair damaged proteins and alleviate ER stress through UPR.

If the misfolded protein cannot be repaired, ERAD can directly degrade the low-quality proteins through the ubiquitin proteasome system (UPS) to avoid ER stress and the release of defective proteins (Li et al., 2017; Lemmer et al., 2021). This study showed that UPS-related genes such as *UBE2*, *UBE3*, *TRIM63*, etc. (Figure 6C) were significantly upregulated in the whole brain region after heat stress, indicating significant UPS-mediated ERAD activation. Moreover, the FB exhibited pronounced enrichment of ubiquitination-mediated proteolysis (Figure 5B), underscoring its role as the ERAD regulatory hub. It is noteworthy that the coordination between HSPs and ubiquitin ligase can facilitate the removal of misfolded proteins through ERAD (Matsumura et al., 2013).

Therefore, this study suggests that abnormal proteins may be removed through a synergistic regulatory mechanism of UPR repair and ERAD degradation to maintain ER homeostasis and mitigates cytotoxicity in *L. longirostris* under thermal stress.

### 4.4 Under heat stress, lipid metabolism is inhibited

The normal metabolic activity of fish is highly dependent on the environment temperature, and temperature variation beyond a certain range will cause physiological disorders and eventually become toxic to fish (Fang et al., 2023; Alfonso et al., 2021). In this study, global downregulation of lipid metabolism genes (*FSN*, *ACADM*) and PPAR signaling were also observer in the SC region (Figures 7C, D), suggesting that lipid metabolism is inhibited, which may be a strategy to reduce energy consumption in *L. longirostris*.

The ER plays an important role in lipid synthesis, transport and lipid droplet formation in cells (Deng et al., 2023). The ER dysfunction is a key driver of abnormal lipid metabolism (Celik et al., 2023). In this study, heat stress not only induced ER stress in FB region, but also led to lipid metabolism disorder in SC region. This indicates that ER stress in the FB region and metabolic suppression in the SC region may collectively counteract heat stress-induced energy imbalance through cross-regional synergistic mechanisms, thereby maintaining systemic physiological homeostasis.

Regrettably, although this study has systematically analyzed for the first time the division of labor of different brain regions of *L. longirostris* in response to heat stress, there are still potential limitations in the research results. On the one hand, the study only involves a 24 h acute high-temperature stress, which fails to reflect the impacts of chronic heat stress. On the other hand, whether there is an interaction between the inhibition of lipid metabolism in the SC region and the acute ER stress in the FB region remains unclear. In the follow-up research, we will combine acute and chronic models to comprehensively evaluate the dynamic responses to heat stress. Meanwhile, by integrating proteomics and metabolomics, we will further conduct an in-depth analysis of the mechanisms by which heat stress affects different brain regions of *L. longirostris*.

## 5 Conclusion

This study has for the first time revealed the differential response mechanisms of five brain regions (OB, FB, MB, CB and SC) of the *L. longirostris* to heat stress. Specifically, the FB region clears abnormal proteins by activating ER stress, while the SC region reduces energy consumption by inhibiting lipid metabolism. These two regions work synergistically to maintain homeostasis under heat stress. Our findings provide new insights into heat-adaptation mechanism of *L. longirostris* and lays the foundation for the research on fish neurometabolic diseases. Future studies need to integrate multi-omics data to explore the interaction mechanism between ER stress and lipid metabolism, and explore chronic stress models, so as to further promote the in-depth analysis of heat stress mechanisms and provide a theoretical basis for the protection of fish resources under climate change.

## Data Availability

The datasets presented in this study can be found in online repositories. The names of the repository/repositories and accession number(s) can be found in the article/Supplementary Material.

## Supplementary material

SUPPLEMENTARY TABLE S1 Sequencing data quality table.

SUPPLEMENTARY TABLE S2 The results of mapping comparison with reference genomes.

SUPPLEMENTARY FIGURE S1 GO enrichment and classification.

SUPPLEMENTARY FIGURE S2 KEGG enrichment and classification.

SUPPLEMENTARY FIGURE S3 WGCNA module clustering.

SUPPLEMENTARY FIGURE S4 k04141 (Endoplasmic reticulum Protein Processing) in the FB region.
