# Supplementary figures and images for: RNA-seq revealed the effects of heat stress on different brain regions of Leiocassis longirostris
Source: Front Physiol. 2025 May 13;16:1579499. doi: 10.3389/fphys.2025.1579499 (PMC12106027; doi:10.3389/fphys.2025.1579499)

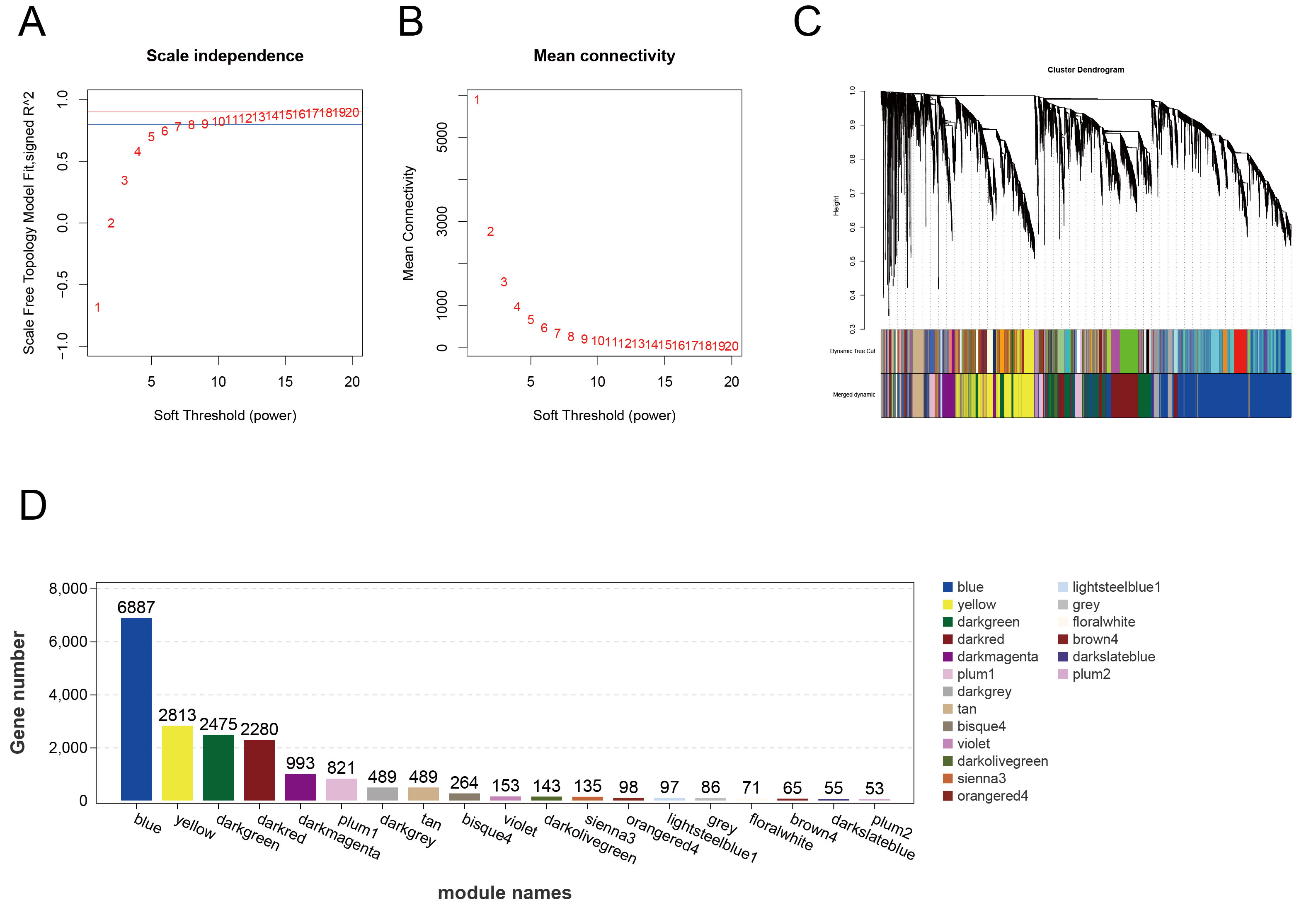

Supplement: Supplementary file 2 [file Image3.jpeg]

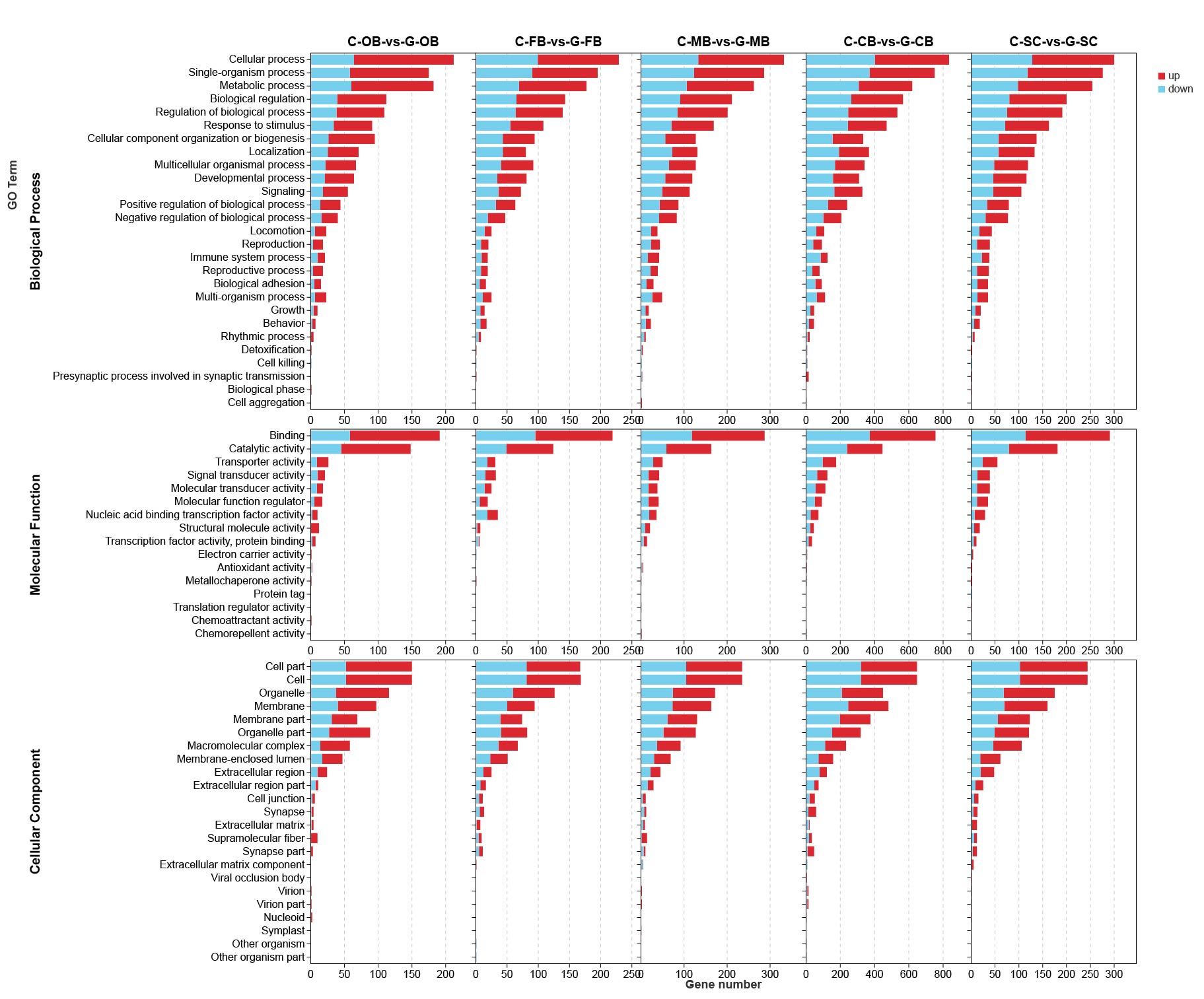

Supplement: Supplementary file 3 [file Image1.jpeg]

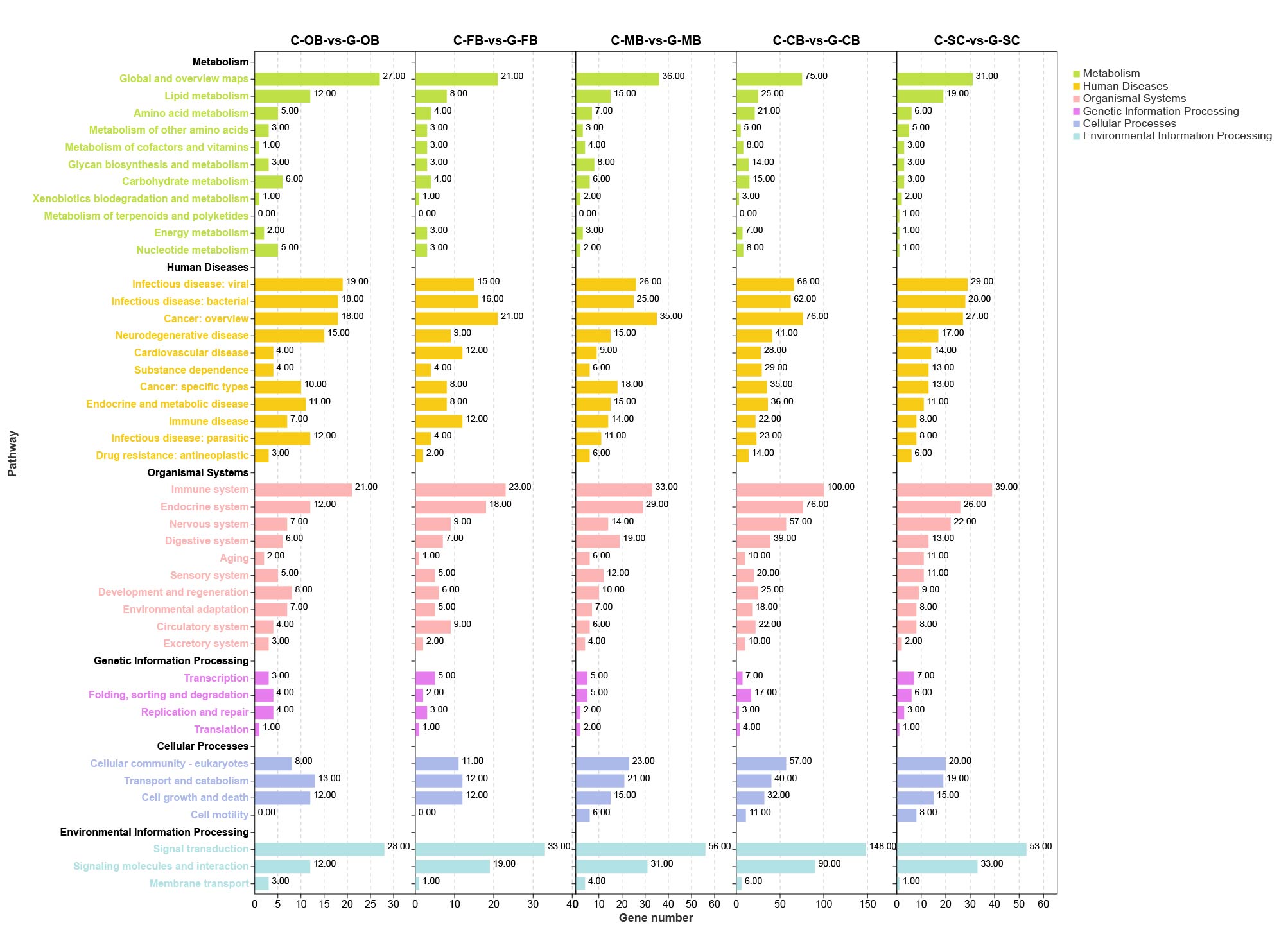

Supplement: Supplementary file 4 [file Image2.jpeg]

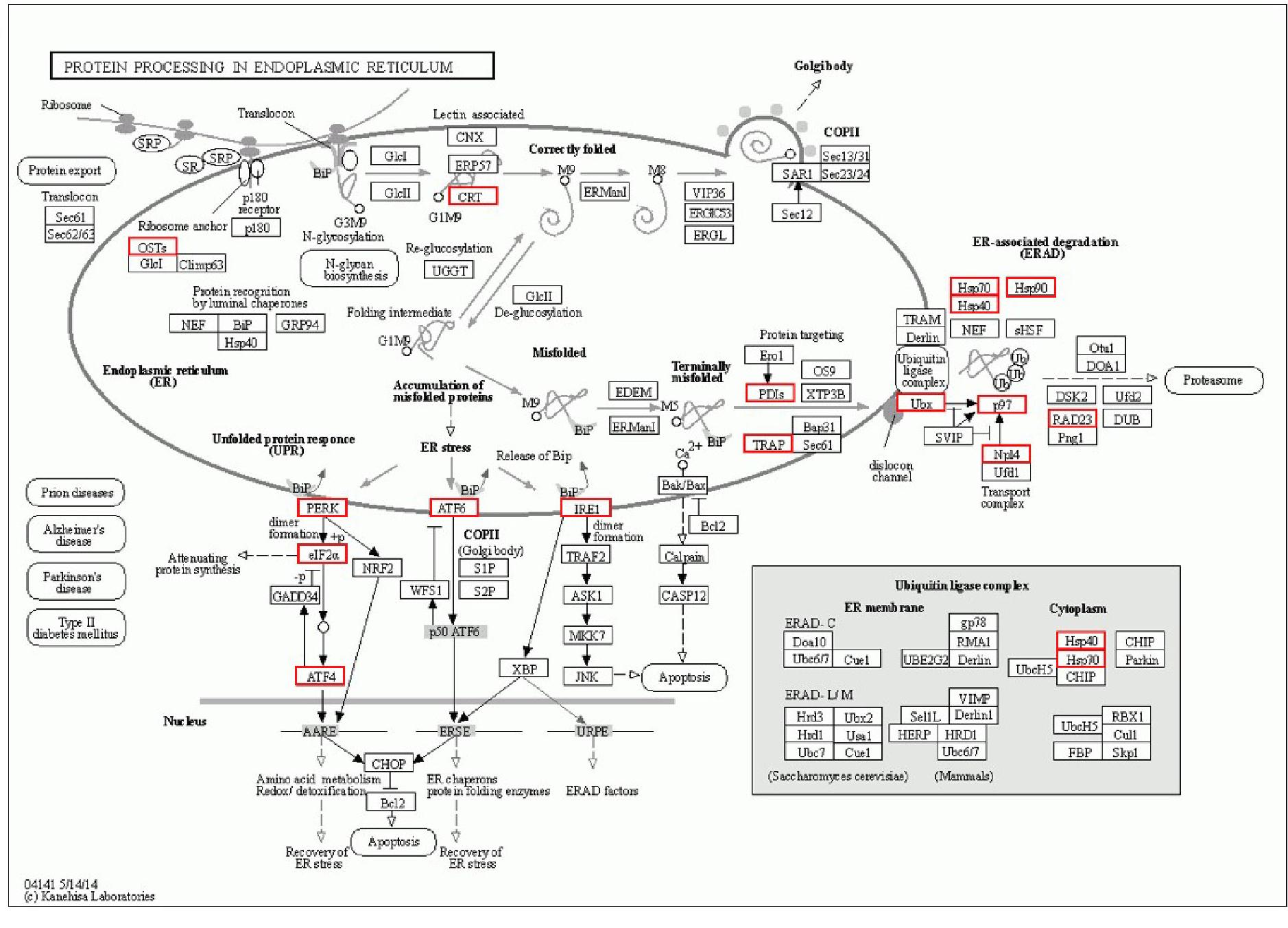

Supplement: Supplementary file 6 [file Image4.jpg]
